# The epileptic heart: Let's not forget to look at the atria!

**DOI:** 10.1111/epi.18650

**Published:** 2025-09-23

**Authors:** Guilherme L. Fialho, Ramsés Miotto, Katia Lin

**Affiliations:** ^1^ Cardiology Division Federal University of Santa Catarina Florianopolis Brazil; ^2^ Medical Sciences Postgraduate Program Federal University of Santa Catarina Florianopolis Brazil; ^3^ Center for Applied Neurosciences Federal University of Santa Catarina Florianopolis Brazil; ^4^ Neurology Division Federal University of Santa Catarina Florianopolis Brazil

To the Editors,

We read with great interest the study by Duran and Boduroglu^1^ recently published in *Epilepsia*, where the authors report on altered left atrial (LA) anatomy and function in people with epilepsy (PWE) compared to a control group, both without known cardiovascular disease. They found reduced total and active LA emptying fraction, increased passive LA emptying fraction, and increased volumetric index in PWE. By focusing on echocardiographic markers of LA structure and function, the authors provide additional evidence that epilepsy–heart interactions extend beyond transient peri‐ictal changes to include chronic, measurable alterations in cardiac anatomy.

Recently, our group demonstrated that individuals with temporal lobe epilepsy had subclinical impairments in atrial and ventricular function compared to a control group, despite preserved ejection fraction. In our speckle‐tracking echocardiography study, left and right ventricular longitudinal strain, LA biplanar contractile strain, and right atrial reservoir, conduit, and contractile strain were reduced in PWE.^2^ Additionally, epilepsy was a predictor of reduced contractile and reservoir strain values of both atria. LA reservoir strain has been recently incorporated as a tool for the evaluation of diastolic function and for the diagnosis of heart failure with preserved ejection fraction.^3^, ^4^

Together, these data suggest that functional myocardial changes and structural LA enlargement may represent different stages of a shared pathological process. Potential drivers include chronic autonomic imbalance, systemic inflammation, microvascular dysfunction, and seizure‐related myocardial injury, each of which has been implicated in epilepsy‐associated cardiac alterations.^5^, ^6^, ^7^

From an arrhythmogenic perspective, LA structural remodeling is a well‐recognized substrate for conduction disturbances and atrial fibrillation (AF). Desai et al.,^8^ analyzing >1.4 million US epilepsy hospitalizations, found arrhythmias in nearly one third of cases, with AF being the most frequent. Previously, we demonstrated that individuals with chronic epilepsy have markedly elevated P‐wave heterogeneity, comparable to patients with AF who were 20 years older, reflecting significant atrial electrical dispersion even in the absence of overt arrhythmia.^5^ Beyond AF, the presence of LA enlargement and functional impairment are well‐established predictors of stroke and mortality in the general population, which could further explain the increased mortality risk in PWE.^9^

By identifying LA structural remodeling in this context, Duran and Boduroglu^1^ add a valuable piece to this multidisciplinary puzzle. Future prospective studies should clarify whether LA enlargement in epilepsy predicts incident arrhythmias or adverse cardiovascular outcomes, and whether its progression can be attenuated through optimized seizure control, cardiovascular risk management, or targeted arrhythmia surveillance. Integration of volumetric, deformation, and electrophysiological markers into epilepsy care may ultimately enable earlier identification of high‐risk individuals and more personalized preventive strategies.

## AUTHOR CONTRIBUTIONS

Guilherme L. Fialho designed and wrote this letter. Guilherme L. Fialho, Ramsés Miotto, and Katia Lin conceptualized and revised the letter.

## CONFLICT OF INTEREST STATEMENT

K.L. holds a National Council for Scientific and Technological Development–CNPq PQ1C Research Fellowship (process number 306916/2023‐1). R.M. and G.L.F. have no conflicts of interest. We confirm that we have read the Journal's position on issues involved in ethical publication and affirm that this report is consistent with those guidelines.

## Data Availability

All data supporting the findings of this letter are available from the corresponding author upon request.
